# Discovery of E2730, a novel selective uncompetitive GAT1 inhibitor, as a candidate for anti‐seizure medication

**DOI:** 10.1002/epi4.12741

**Published:** 2023-05-18

**Authors:** Kazuyuki Fukushima, Hiroyuki Higashiyama, Yuji Kazuta, Keisuke Hashimoto, Naoto Watanabe, Yoshiaki Furuya, Yoshimasa Ito, Ting Wu, Takashi Kosasa, Delia M. Talos, Yeri Song, Nicholas S. Roberts, Frances E. Jensen, Takahisa Hanada, Katsutoshi Ido

**Affiliations:** ^1^ Deep Human Biology Learning Eisai Co., Ltd. Tsukuba Ibaraki Japan; ^2^ Neurology Business Group Eisai Co., Ltd. Tsukuba Ibaraki Japan; ^3^ Alzheimer's Disease and Brain Health Eisai Co., Ltd. Tsukuba Ibaraki Japan; ^4^ Department of Neurology, Perelman School of Medicine University of Pennsylvania Philadelphia Pennsylvania USA

**Keywords:** anti‐seizure medications, GABA transporters, GAT1, uncompetitive inhibition

## Abstract

**Objective:**

As of 2022, 36 anti‐seizure medications (ASMs) have been licensed for the treatment of epilepsy, however, adverse effects (AEs) are commonly reported. Therefore, ASMs with a wide margin between therapeutic effects and AEs are preferred over ASMs that are associated with a narrow margin between efficacy and risk of AEs. E2730 was discovered using in vivo phenotypic screening and characterized as an uncompetitive, yet selective, inhibitor of γ‐aminobutyric acid (GABA) transporter 1 (GAT1). Here, we describe the preclinical characteristics of E2730.

**Methods:**

Anti‐seizure effects of E2730 were evaluated in several animal models of epilepsy: corneal kindling, 6 Hz–44 mA psychomotor seizure, amygdala kindling, Fragile X syndrome, and Dravet syndrome models. Effects of E2730 on motor coordination were assessed in accelerating rotarod tests. The mechanism of action of E2730 was explored by [^3^H]E2730 binding assay. The GAT1‐selectivity over other GABA transporters was examined by GABA uptake assay of GAT1, GAT2, GAT3, or betaine/GABA transporter 1 (BGT‐1) stably expressing HEK293 cells. To further investigate the mechanism for E2730‐mediated inhibition of GAT1, in vivo microdialysis and in vitro GABA uptake assays were conducted under conditions of different GABA concentrations.

**Results:**

E2730 showed anti‐seizure effects in the assessed animal models with an approximately >20‐‍fold margin between efficacy and motor incoordination. [^3^H]E2730 binding on brain synaptosomal membrane was abolished in GAT1‐deficient mice, and E2730 selectively inhibited GAT1‐mediated GABA uptake over other GABA transporters. In addition, results of GABA uptake assays showed that E2730‐mediated inhibition of GAT1 positively correlated to the level of ambient GABA in vitro. E2730 also increased extracellular GABA concentration in hyperactivated conditions but not under basal levels in vivo.

**Significance:**

E2730 is a novel, selective, uncompetitive GAT1 inhibitor, which acts selectively under the condition of increasing synaptic activity, contributing to a wide margin between therapeutic effect and motor incoordination.

**Key points**

- E2730 was discovered by in vivo phenotypic screening as an ASM candidate with a wide margin between therapeutic and adverse effects.
- GAT1 was identified as the target of E2730 according to [^3^H]E2730 binding assay in GAT1‐deficient mouse brain.
- E2730‐mediated inhibition of GAT1 positively correlated to ambient GABA levels, suggesting the uncompetitive inhibition mode of E2730.
- The wide margin between efficacy and motor incoordination of E2730 can be attributed to its selectivity to increased synaptic activity.

## 1 INTRODUCTION

Epilepsy, one of the most common and disabling chronic neurological disorders, affects approximately 1% of the general population. As of 2022, 36 anti‐seizure medications (ASMs) have been licensed for the treatment of epilepsy worldwide.^1^ However, 30% of patients with epilepsy continue to experience seizures despite treatment with ASMs.^2^ In addition to achieving seizure freedom, lowering the risk of adverse effects (AEs) is an important objective for new ASMs. Up to 88% of people with epilepsy experience AEs following treatment with ASMs, such as dizziness and sedation, which can negatively impact patients' quality of life (QoL) and result in intolerability.^3^, ^4^ The efficacy and tolerability profiles of currently available ASMs warrant continuing efforts to discover and develop next‐generation ASMs with improved safety profiles and seizure control.^5^ Indeed, more than 30 ASM candidates are being investigated in the preclinical and clinical development pipeline, and the mechanisms of action (MOAs) of some candidates are distinct.

Given its complexity, people with epilepsy could experience multiple types of seizures, ranging from non‐convulsive to convulsive seizures, or from focal to generalized seizures. Appropriate selection of ASMs based on their MOA is key to achieving optimal treatment outcomes.^6^ E2730 was discovered by in vivo phenotypic screening in epilepsy and rotarod mouse models, which aimed to identify ASM candidates with a broad dosing margin between therapeutic response and AEs, and was investigated in animal models of epilepsy. Following the discovery of E2730, we report the MOA of E2730 and its preclinical profile, i.e. therapeutic response and AEs in animal models of epilepsy.

## 2 MATERIALS AND METHODS

### 2.1 Anti‐seizure effects in animal models of epilepsy

All animal experiments were performed in compliance with the regulations of the Animal Ethical Committee of Eisai Co., Ltd. or the Institutional Animal Care and Use Committee Office of Animal Welfare of the University of Pennsylvania. Animals were kept at approximately 23°C (permitted range: 20–26°C) in a relative humidity of 55% (permitted range: 40%–70%) with a 12‐hour dark/light cycle (lighting between 07:00 and 19:00).

#### 2.1.1 Corneal kindling in mice

A corneal kindling mouse model was developed following the method described by Potschka and Löscher.^7^ Male C57BL/6N mice (5 weeks old; Charles River Laboratories Japan, Japan) were stimulated using corneal electrodes (4 mA, 50 Hz, 3‐second duration each) twice daily for 10 consecutive days, and stimulated mice were observed and rated based on the Racine's scale (0, no seizure; 1, mild facial clonus and eye blinking; 2, severe facial clonus, head nodding, or chewing; 3, unilateral or alternating forelimb clonus; 4, bilateral forelimb clonus and rearing; 5, bilateral forelimb clonus with rearing and falling). Mice with scores ≥3 were deemed epileptic, whereas mice with scores <3 were deemed non‐epileptic. Mice rated 4 or 5 (i.e. fully kindled mice) were used for drug evaluation. E2730 (5–50 mg/kg) or vehicle (0.45% methylcellulose solution containing 10% dimethyl sulfoxide [DMSO] and 4.5% cremophor) was administered orally to the kindled mice 1 hour before the electrical stimulation.

#### 2.1.2 Pharmaco‐resistant 6 Hz–44 mA psychomotor seizures in mice

Psychomotor seizures were stimulated by applying currents (44 mA, 6 Hz, 0.2 millisecond rectangular pulse width, 3‐second duration each) via a corneal electrode (SEN‐7203; Nihon Kohden). E2730 (5–50 mg/kg), levetiracetam (Tokyo Chemical Industry; 20–200 mg/kg), or vehicle was orally administered to ddY mice (male, 5 weeks old; Japan SLC Inc.) 1 hour before electrostimulation. Stimulated mice were observed for the presence or absence of seizures for 30 second. Behavioral seizures observed in mice were classified into the following four types: stun, forelimb clonus, twitching of the vibrissae, and Straub‐tail. A mouse demonstrating one or more of these four behaviors was defined as epileptic.

#### 2.1.3 Amygdala kindling in rats

A tripolar electrode was implanted into the basolateral amygdala of Wistar‐Kyoto rats (male, 7 weeks old; Charles River Laboratories Japan, Japan). After at least 5 days of recovery, the amygdala was stimulated using an electronic stimulator at currents starting at 0.04 mA (50 Hz, 1 millisecond monophasic square‐wave pulses, 1‐second duration each) then increasing by 25% to a 30‐second duration until the after‐discharge threshold (ADT) was attained. ADT was defined as the current at which abnormal electroencephalogram and at least Racine's stage‐1 seizure behavior were observed. In the Racine's stage, seizure severity was classified as: 1, mouth and facial movements; 2, head nodding; 3, forelimb clonus; 4, rearing; 5, rearing and falling. From the next day onwards, electrical stimulation at the previously determined ADT was performed once daily in each rat. The rats showing ≥3 consecutive Racine stage‐5 seizures were deemed epileptic and used for drug evaluation. The pre‐ADT (i.e. baseline) seizure threshold of each rat was established prior to drug administration. After >1 hour following the determination of pre‐ADT seizure threshold, E2730 (5–50 mg/kg), levetiracetam (25 mg/kg), or vehicle was orally administered to the rats. One hour after the administration, the post‐ADT seizure threshold of each rat was ascertained, and the ratios of post‐ADT to pre‐ADT readings were analyzed.

#### 2.1.4 Audiogenic seizures in *Fmr1* knockout mice model of fragile X syndrome

Fragile X syndrome (FXS) is the most common single‐gene cause of autism spectrum disorder, typically due to mutations in the *FMR1* gene; 10–18% of patients with FXS experience seizures.^8^ The *Fmr1* knockout (KO) mouse is an established animal model for FXS. E2730 (2.5–300 mg/kg) or vehicle was orally administered to *Fmr1* KO mice (male, 20–27 days postnatal; Jackson Laboratory) 1 hour prior to drug evaluation. The mice were placed in a sound‐attenuating acoustic chamber and acclimated for 5 minutes and exposed to an audio stimulation (110 dB, 60‐second duration), and the behavior of the stimulated mice was video recorded and monitored for tonic–clonic seizures.

#### 2.1.5 Hyperthermia‐induced seizures in *Scn1a* ^+/−^ mouse model of Dravet syndrome

*Scn1a* heterozygous mutant (*Scn1a* ^+/−^) mice, a model of Dravet Syndrome (DS), and the littermate wild‐type (*Scn1a* ^+/+^) mice (male, 24–28 days postnatal; Jackson Laboratory, US) received E2730 (10, 20 mg/kg) or vehicle orally 1 hour before hyperthermia stimulation. A rectal temperature probe (Physitemp, NJ) was fitted at 50 minutes post‐treatment, and mice were acclimated to the probe and test chamber for 10 minutes. At 1‐hour post‐treatment, core body temperature was elevated by a heat lamp connected to a feedback temperature controller (Physitemp) by 0.5°C every 2 minutes until a maximum of 42.5°C was reached. The threshold body temperatures for generalized tonic–clonic seizures were recorded for each mouse. The cut‐off time was set as 180 seconds.

#### 2.1.6 Rotarod test in mice

Motor incoordination is one of the most common AEs associated with the use of ASMs.^9^ Therefore, motor coordination was evaluated in mice receiving E2730 to assess if it is a new ASM candidate with a wide margin between therapeutic effects and AEs. E2730 (50–400 mg/kg) or vehicle was orally administered in ddY mice (male, 5 weeks old) 1 hour before the rotarod test, and the latency for mice to fall off the rod was measured. The rod rotation of the rotarod apparatus (Muromachi Kikai) was continuously accelerated from 0 to 40 rpm in a cut‐off time of 180 seconds.

### 2.2 Radiolabeled E2730 binding assay

All experiments using human samples were performed in compliance with the regulations of the Research Ethical Committee of Eisai Co., Ltd.

#### 2.2.1 Saturation binding of [ ^3^H]E2730 in rat and human brain synaptosomal membranes

Synaptosomal membranes were prepared using the whole brain without cerebellum, brainstem, or olfactory bulb from Sprague Dawley rats (male, 7 weeks old) or human cerebral cortex based on Fuks et al.^10^ and Hanada et al.^11^ Synaptosomal membranes (0.4 mg protein/tube) were incubated with 9.8–5000 nM [^3^H]E2730 for 120 minutes at 4°C. Non‐specific binding was defined as the residual binding observed in the presence of 1 mM unlabeled E2730. At the end of the incubation period, the synaptosome‐bound radioligand was recovered by rapid filtration through Whatman GF/B glass fiber filters pre‐soaked in 0.15% polyethyleneimine using a 24‐well cell harvester (Brandel). The filters were washed three times with ice‐cold 50 mM Tris–HCl buffer (pH 7.4) and incubated overnight in a scintillator (PerkinElmer), then the radioactivity was measured by a liquid scintillation counter (PerkinElmer).

#### 2.2.2 Competitive displacement of [ ^3^H]E2730 binding with ASMs

For the competitive displacement assay, rat synaptosomal membrane extract (0.2 mg protein/tube) was incubated for 120 minutes at 4°C with 20 nM [^3^H]E2730 and the following ASMs: carbamazepine (FUJIFILM Wako Pure Chemical Corporation; 100 μM); lamotrigine (Sigma‐Aldrich, MO; 300 μM); diazepam (FUJIFILM Wako Pure Chemical Corporation; 10 μM); gabapentin (Tokyo Chemical Industry; 300 μM); ethosuximide (Tokyo Chemical Industry; 3000 μM); perampanel (Eisai Co., Ltd.; 30 μM), retigabine (LKT Laboratories; 100 μM); levetiracetam (Tokyo Chemical Industry; 1000 μM); acetazolamide (Sigma‐Aldrich; 300 μM); sodium valproate (FUJIFILM Wako Pure Chemical Corporation; 3000 μM); zonisamide (FUJIFILM Wako Pure Chemical Corporation; 300 μM); and topiramate (Sigma‐Aldrich; 300 μM). Unlabeled E2730 (0.1–10 μM) was also assessed to confirm a representative displacement. Non‐specific binding was defined as the residual binding observed in the presence of 1 mM unlabeled E2730. Radioactivity of the synaptosome‐bound radioligand was measured in the same way as described above.

#### 2.2.3 [ ^3^H]E2730 binding in GAT1‐deficient mouse brain

Synaptosomal membrane extract was prepared from homozygous γ‐aminobutyric acid (GABA) transporter 1 (GAT1) KO (Homo), heterozygous GAT1 KO (Hetero), and the littermate wild‐type (WT) mice (male, 10 weeks old) using the same approach as described above. Synaptosomal membrane extract (0.4 mg protein/tube) was incubated with 200 nM [^3^H]E2730 for 120 minutes at 4°C. Non‐specific binding was defined as the residual binding observed in the presence of 1 mM unlabeled E2730. The radioactivity of the synaptosome‐bound radioligand was measured in the same way as described above.

### 2.3 Radiolabeled GABA uptake assay

#### 2.3.1 Subtype selectivity of E2730 inhibitory effect among GABA transporters

HEK293 cells stably expressing human GAT1 (hGAT1), GAT2 (hGAT2), GAT3 (hGAT3), or betaine/GABA transporter 1 (hBGT‐1) were plated onto 96‐well microtiter plates at a density of 4.0 × 10^4^ cells/well in Dulbecco's Modified Eagle's Medium (DMEM, containing 10% fetal bovine serum, 100 units/mL penicillin, 100 μg/mL streptomycin, 1% non‐essential amino acid solution, and 25 mM HEPES) and incubated overnight at 37°C in a 5% CO_2_ atmosphere. After washing the cells with Hanks’ balanced salt solution (HBSS, containing 140 mg/L CaCl_2_, 100 mg/L MgCl_2_, 20 mM HEPES, and 10 mM NaOH), E2730 (0.0128–1000 μM) was added to the plate. Following the addition of 50 μM [^3^H]GABA, cells were incubated for 5 minutes at 37°C in a 5% CO_2_ atmosphere and then washed with cold HBSS. Washed cells were incubated in a scintillator for 1 hour at room temperature, followed by radioactivity measurements using a liquid scintillation counter.

#### 2.3.2 E2730‐mediated inhibition of GABA uptake under different GABA concentrations

hGAT1 stably expressing HEK293 cells were plated onto 96‐well microtiter plates at a density of 4.0 × 10^4^ cells/well in DMEM and incubated overnight at 37°C in a 5% CO_2_ atmosphere. After washing the cells with assay buffer, E2730 (0.0128–1000 μM), tiagabine (Tokyo Chemical Industry; 0.000256–100 μM), or vehicle was added to the plate in the presence of 1% DMSO. After adding [^3^H]GABA (0.01–500 μM), the plate was incubated for 5 minutes at 37°C in a 5% CO_2_ atmosphere. The radioactivity of the treated cells was assessed following the same method described above.

### 2.4 Measurement of extracellular GABA concentration in mouse hippocampus

A guide cannula was implanted in the left hippocampus (2.7 mm posterior, 3.3 mm left, and 1.8 mm below from the bregma) of ddY mice (male, 5 weeks old). After 5–7 days of recovery, a microdialysis probe (Eicom) was inserted into the left hippocampus through the guide cannula. The tip of the microdialysis probe reached 4.8 mm below the bregma. The next day, the probe was connected to a perfusion tube and perfused with Ringer solution (containing 147.0 mM NaCl, 4.7 mM KCl, 0.6 mM MgSO_4_, 2.5 mM CaCl_2_, 5.0 mM HEPES, pH 7.4) at a flow rate of 1.5 μL/min. After ≥160 minutes of perfusion, collection of the perfusate (30 μL/20 min/fraction) was initiated, and 12 fractions per animal were collected for 240 minutes. E2730 (10–100 mg/kg), tiagabine (3–30 mg/kg), or vehicle was orally administered 72 minutes after initiating sample collection. Tiagabine was used as a reference ASM of a non‐competitive GAT1 inhibitor. Perfusate was switched from Ringer solution to high‐potassium Ringer solution (51.7 mM NaCl, 100.0 mM KCl, 0.6 mM MgSO_4_, 2.5 mM CaCl_2_, 5.0 mM HEPES, pH 7.4) after 127 minutes following the collection. GABA concentration in each sample was measured by a high‐performance liquid chromatography with tandem mass spectrometry method.

### 2.5 Statistical analysis

Data are expressed as the mean ± standard error of the mean. GraphPad Prism (GraphPad Software) or SAS (SAS Institute Japan) was used for statistical analyses. A *P*‐value of <0.05 was considered statistically significant.

## 3 RESULTS

### 3.1 E2730 is a novel ASM candidate with a wide margin between therapeutic effect and adverse effect

**FIGURE 1:**
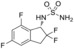
Chemical Structure of E2730.

We characterized the pharmacologic profile of E2730 (Figure 1) in corneal kindling mice, a well‐established model of focal epilepsy for drug screening and development. The 50% effective dose (ED_50_) of E2730 was 7.9 mg/kg in the corneal kindling mice, and the 50% toxic dose (TD_50_) was 350 mg/kg in the accelerating rotarod test (Table 1). The protective index (i.e. the ratio of TD_50_ to ED_50_) of E2730 was 44.3.

**TABLE 1:** Anti‐seizure and motor‐incoordination characteristics of E2730 and selected ASMs.

Compound	ED_50_ (95% CI) in corneal kindling mice, mg/kg	TD_50_ (95% CI) in mouse rotarod test, mg/kg	Protective index (i.e., TD_50_/ED_50_)
E2730	7.9 (4.4, 12)	350 (310, 450)	44.3
Tiagabine	0.51^a^	1.29^b^	2.5
Carbamazepine	9^c^ (5, 11)	45.4^b^	5.0
Valproate	110^c^ (83, 143)	398^b^	3.6
Phenobarbital	11^c^ (7, 15)	69^b^	6.3
Clonazepam	0.04^c^ (0.02, 0.07)	0.26^b^	6.5
Ethosuximide	>254^c^	341^b^	<1.3
Levetiracetam	16^c^ (6, 43)	>500^b^	>31.2

Abbreviations: ASM, anti‐seizure medication; CI, confidence interval; ED_50_, 50% effective dose; TD_50_, 50% toxic dose.

^a^ Data from Madsen et al.^38^

^b^ Data from Levy et al.^39^

^c^ Data from Matagne et al.^40^

### 3.2 Profiling of E2730 anti‐seizure effects in epilepsy animal models

**FIGURE 2:**
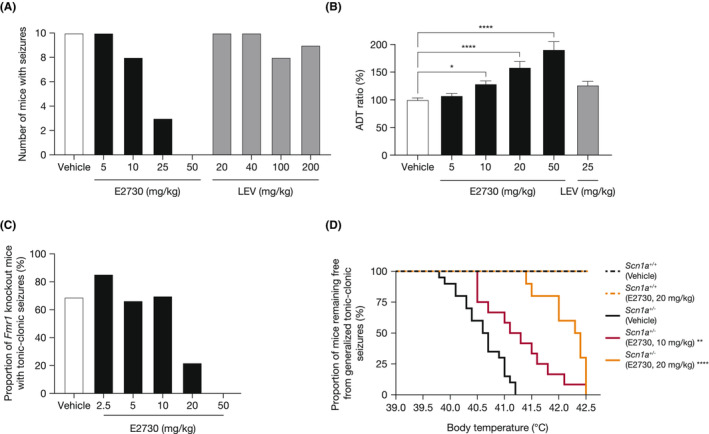
Profiling of anti‐seizure effect of E2730 in animal epilepsy models. (A) Anti‐seizure effects of E2730 and levetiracetam on 6 Hz–44 mA psychomotor seizures in mice. Data represent number of animals which showed at least one behavioral seizure (each *n* = 10). ED_50_ and 95% CI of each compound were determined using probit analysis. (B) Anti‐seizure effects of E2730 and levetiracetam in rat amygdala kindling model. Data represent the mean ± SEM (*n* = 11–12). ADT ratio was the ratio of post‐ADT to pre‐ADT. Differences between the E2730‐ or LEV‐ vs vehicle‐treated groups were analyzed using the Kruskal Wallis test followed by the Dunn's multiple comparison test. (C) Anti‐seizure effects of E2730 on audiogenic seizures in *Fmr1* knockout mice. Data represent the percentages of *Fmr1* mice exhibiting tonic–clonic seizures (vehicle, *n* = 26; 2.5–300 mg/kg, *n* = 6–11). The ED_50_ and 95% CIs were calculated by nonliner regression following normalization to seizure occurrence in vehicle‐treated group. (D) Anti‐seizure effects of E2730 on hyperthermia‐induced seizures in *Scn1a* ^+/−^ mice. The Kaplan–Meier analysis for generalized tonic–clonic seizures. Each line shows percentage of mice remaining seizure‐free at the indicated body temperatures (*n* = 8–20). *P < 0.05, **P < 0.01, and ****P < 0.0001 (vs *Scn1a* ^+/−^ [Vehicle] group, log‐rank tests). ADT, after‐discharge threshold; CI, confidence interval; ED_50_, 50% effective dose; LEV, levetiracetam; post‐ADT, ADT determined after drug administration; pre‐ADT, ADT determined prior to drug administration

The anti‐seizure effect of E2730 against multiple seizure types was further investigated in other epilepsy animal models, such as 6 Hz‐44 mA psychomotor seizures in mice, amygdala kindling rats, *Fmr1* KO mice, and *Scn1a* ^+/−^ mice. E2730 was associated with dose‐dependent decreases in seizure frequency in these models. The ED_50_ of E2730 in the 6 Hz‐44 mA psychomotor seizure mice was 17 mg/kg (Figure 2A). In the amygdala kindling rats, E2730 significantly increased the post‐ADT/pre‐ADT ratios by 129%, 159%, and 191% at 10, 20, and 50 mg/kg, respectively (Figure 2B). The ED_50_ of E2730 for tonic–clonic seizures was 17.1 mg/kg in the audio‐stimulated *Fmr1* KO mice (Figure 2C). In *Scn1a* ^+/−^ mice, the median threshold body temperature for generalized tonic–clonic seizures was significantly higher with E2730 (10 mg/kg, 41.2°C; 20 mg/kg, 42.4°C) compared with vehicle (40.7°C) (Figure 2D). No seizures occurred in the littermate WT (*Scn1a* ^+/+^) mice at a maximum temperature of 42.5°C, irrespective of treatment with vehicle or E7230 20 mg/kg.

### 3.3 The pharmacology and MOA of E2730

**FIGURE 3:**
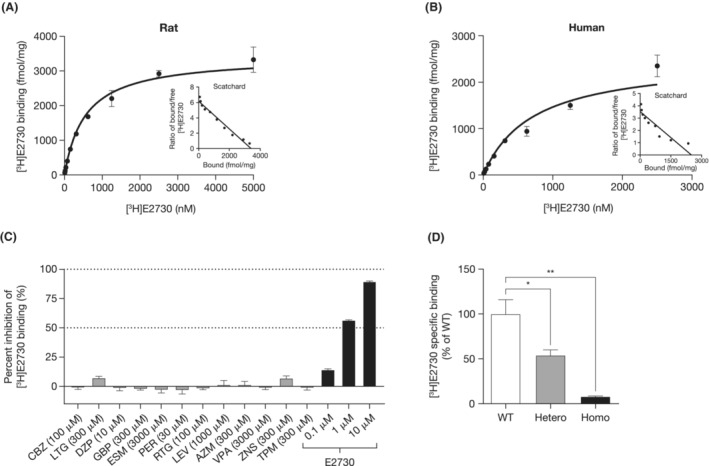
[ ^3^H]E2730 binding assay in mouse, rat, or human brain. (A) Saturation binding of [^3^H]E2730 in rat brain synaptosomal membranes. Data represent the mean ± SEM from three independent experiments. Inset represents the Scatchard plot from the transformed data. The *B*_max_ and *K*_D_ values were calculated based on the Scatchard plot. (B) Saturation binding of [^3^H]E2730 in human brain synaptosomal membranes. Data represent the mean ± SEM from three independent experiments. Inset represents the Scatchard plot from the transformed data. (C) Competitive displacement of [^3^H]E2730 binding in rat brain synaptosomal membranes with ASMs. Data represent the mean ± SEM from three independent experiments. (D) [^3^H]E2730 binding in brain synaptosomal membranes of GAT1‐deficient mice. Data represent the mean ± SEM (*n* = 3). *P < 0.05, **‍P < 0.01 (vs WT group, Dunnett multiple comparison test). ASM, anti‐seizure medication; AZM, acetazolamide; *B*_max_, maximum binding; CBZ, carbamazepine; DZP, diazepam; ESM, ethosuximide; GABA, γ‐aminobutyric acid; GAT1, GABA transporter 1; GBP, gabapentin; Homo, homozygous GAT1‐deficient mice; Hetero, heterozygous GAT1‐deficient mice; *K*_D_, binding affinity; LEV, levetiracetam; LTG, lamotrigine; PER, perampanel; RTG, retigabine; SEM, standard error of mean; TPM, topiramate; VPA, valproate; WT, the littermate wild‐type mice; ZNS, zonisamide.

The binding affinity of E2730 on brain synaptosomal membranes was assessed by the maximum binding capacity (*B*_max_) and equilibrium dissociation constant (*K*_D_), using the radiolabeled [^3^H]E2730. The *B*_max_ and *K*_D_ of E2730 on synaptosomal membranes were 3419 fmol/mg protein and 553.4 nM, respectively, in rats (Figure 3A), and 2503 fmol/mg protein and 709.9 nM for humans (Figure 3B).

Competitive displacement assays were conducted to determine whether the binding site of E2730 was different from those of clinically available ASMs. Unlabeled E2730 displaced the radiolabeled [^3^H]E2730 in a concentration‐dependent manner; conversely, the binding of [^3^H]E2730 was not displaced by any of the 12 approved ASMs (Figure 3C). We also confirmed that E2730 did not directly affect the activity of human GABA_A_ receptor (GABA_A_R) or voltage‐gated sodium channel (Na_v_) 1.2 (Na_v_1.2), both of which are major targets for approved ASMs (Figures S1 and S2). In addition, no significant binding of E2730 was observed in the binding assay against a panel of 86 receptors, transporters, and ion channels contributing to physiological functions (Table S1).

By differential proteomics,^12^ GAT1 was identified as the most probable target of E2730. To validate it, the proportion of [^3^H]E2730 bound to synaptosomal membranes was assessed in GAT1 Hetero and Homo KO mice and compared with that of WT mice. The proportions of bound [^3^H]E2730 were 54.0% (Hetero) and 8.0% (Homo) relative to WT mice (Figure 3D).

**FIGURE 4:**
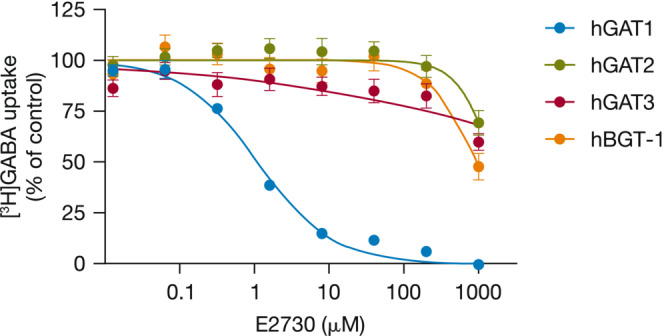
Effects of E2730 on [^3^H]GABA uptake in hGAT1, hGAT2, hGAT3, or hBGT‐1 stably expressing HEK293 cells. Data represent the mean ± SEM from three (hGAT1) or four (hGAT2, hGAT3, and hBGT‐1) independent experiments. GABA, γ‐aminobutyric acid; hGAT1, human GABA transporter 1; hGAT2, human GABA transporter 2; hGAT3, human GABA transporter 3; hBGT‐1, human betaine/GABA transporter 1.

The selectivity of E2730 against different GABA transporter subtypes, GAT and BGT, was assessed based on the changes in GABA uptake in the presence of E2730. The 50% inhibitory concentration (IC_50_) values of E2730 were 1.1 μM for hGAT1, >1000 μM for hGAT2, >1000 μM for hGAT3, and 890 μM for hBGT‐1 (Figure 4).

### 3.4 Mode of E2730‐mediated inhibition of GAT1

**FIGURE 5:**
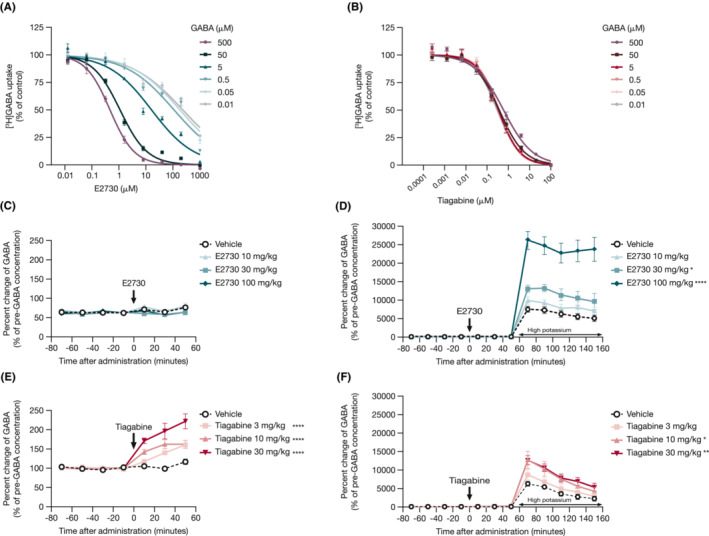
E2730‐mediated inhibition of GAT1 was modulated by ambient GABA concentration. (A) Inhibitory effects of E2730 on [^3^H]GABA uptake in hGAT1 stably expressing cells under different ambient GABA concentrations. Data represent the mean ± SEM from three independent experiments. (B) Inhibitory effects of tiagabine on [^3^H]GABA uptake in hGAT1 stably expressing cells under different ambient GABA concentrations. Data represent the mean ± SEM from three independent experiments. (C) Effect of E2730 on basal extracellular GABA level in the mouse hippocampus. Data represent the mean ± SEM (*n* = 7–8). There was no significant difference between vehicle‐ and E2730‐treated groups. (D) Effect of E2730 on high potassium‐induced increase of extracellular GABA level in the mouse hippocampus. Data represent the mean ± SEM (*n* = 7–8). *P < 0.05, ****P < 0.0001 (vs vehicle‐treated groups at 60–160 min after administration; variance of repeated measurements was analyzed by the Dunnett multiple comparison test after logarithmic transformation of percentages of pre‐GABA concentration). (E) Effect of tiagabine on basal extracellular GABA level in the mouse hippocampus. Data represent the mean ± SEM (*n* = 7–8). ****P < 0.0001 (vs vehicle‐treated groups at 0–60 min after administration; variance of repeated measurements was analyzed by the Dunnett multiple comparison test after logarithmic transformation of percentages of pre‐GABA concentration). (F) Effect of tiagabine on high potassium‐induced increase of extracellular GABA level in the mouse hippocampus. Data represent the mean ± SEM (*n* = 7–8). *P < 0.05, **P < 0.001 (vs vehicle‐treated groups at 60–160 min after administration; variance of repeated measurements was analyzed by the Dunnett multiple comparison test after logarithmic transformation of percentages of pre‐GABA concentration). GABA, γ‐aminobutyric acid; hGAT1, human GABA transporter 1; SEM, standard error of mean.

[^3^H]GABA uptake was assessed in hGAT1 stably expressing cells under different levels of extracellular GABA to characterize the mechanism underlying E2730‐mediated inhibition of GAT1. In the presence of E2730, the inhibition of GAT1 positively correlated with the ambient GABA concentration and deepened with the increasing levels of ambient GABA (Figure 5A), whereas tiagabine inhibited GAT1 with similar potency, irrespective of ambient GABA concentration (i.e. non‐competitive inhibition; Figure 5B). Our results suggest that E2730 inhibits GAT1 in an uncompetitive fashion.

The in vivo effects of E2730‐mediated uncompetitive inhibition of GAT1 on the change in extracellular GABA concentration were evaluated in the mouse hippocampus under basal and high‐potassium‐induced conditions using microdialysis. E2730 did not increase basal extracellular GABA concentration in the mouse hippocampus (Figure 5C) but was associated with a significant increase in the extracellular GABA concentration induced by a high level of potassium (Figure 5D). Conversely, tiagabine significantly increased the extracellular GABA concentration under conditions of basal and high levels of GABA (Figures 5E,F).

## 4 DISCUSSION

AEs of ASMs, such as dizziness and sedation, negatively affect the QoL of people with epilepsy. Motor incoordination is one of the most common AEs of approved ASMs.^9^ The mouse rotarod test was used to assess the impact of ASM candidates on motor incoordination. The corneal kindling mice were used as an ASM screening animal model because it is a cost‐effective tool for focal epilepsy research.^7^

The in vivo phenotypic results showed that E2730 has a higher protective index relative to other ASMs, such as phenobarbital and carbamazepine (Table 1), suggesting that E2730 may be an ASM candidate with a wide margin between therapeutic effect and AEs. Competitive displacement results showed that the binding site of E2730 presents on synaptosomal membranes isolated from mice, rats, and human brains and is distinct from clinically available ASMs, which target the sodium channel, potassium channel, calcium channel, GABA_A_ receptor, α‐amino‐3‐hydroxy‐5‐methyl‐4‐isoxazolepropionic acid (AMPA) receptor, N‐methyl‐D‐aspartate (NMDA) receptor, synaptic vesicle protein 2A (SV2A), or carbonic anhydrase.^13^ The proportions of bound [^3^H]E2730 in GAT1‐deficient mice were significantly lower than that of WT mice, suggesting that GAT1 could be the target of E2730. As high potassium levels, a known ictogenic stimulation, could induce excessive activation of GABA neurotransmission,^14^ we assessed the effects of E2730 on extracellular GABA concentration under basal and hyper‐activated conditions. A high dose of E2730 (up to 100 mg/kg), considering the range of ED_50_ in animal epilepsy models (7.9–17.1 mg/kg), did not affect the extracellular GABA concentration under basal level in the naïve mouse hippocampus but significantly increased the GABA concentration under high‐potassium‐induced hyper‐activation (E2730, 30 and 100 mg/kg). In contrast, tiagabine increased the extracellular GABA concentration under both basal and hyper‐activated conditions. These unique characteristics of E2730 could explain its high protective index: E2730 selectively inhibited GAT1 under hyper‐activated condition without disrupting the physiological excitation and inhibition (E/I) balance, which could lead to undesired sedation. These results also suggest that E2730 inhibits GAT1 in an uncompetitive manner, while tiagabine inhibited GAT1 with similar potency irrespective of ambient GABA concentration (i.e., non‐competitive inhibition as reported previously^15^, ^16^).

Notably, the uncompetitive inhibitor, which is defined as an inhibitor preferentially acting under the condition of higher levels of ligand, is unique among approved drugs with non‐competitive inhibition mode, which is defined as an inhibition pattern showing comparable potency no matter how much ligand is present. For instance, memantine, which is indicated for the treatment of moderate‐to‐severe dementia in Alzheimer's disease (AD), is an uncompetitive antagonist of the *N*‐methyl‐d‐aspartate (NMDA) receptor.^17^ Activation of NMDA receptors under physiological conditions is critical for synaptic plasticity and neuron survival, however, excessive glutamate‐mediated activation of NMDA receptors can cause excitotoxicity and promote cell death,^18^ leading to neurodegeneration associated with AD. Non‐competitive NMDA receptor antagonists, such as ketamine, phencyclidine, and MK‐‍801, have failed in clinical trials for AD due to cognitive disruption and poor tolerability.^19^ However, memantine is associated with potent blockade of the NMDA receptor under pathological conditions (i.e. excessive levels of glutamate), but slight blockade during physiological neurotransmission.^17^ Owing to such uncompetitive characteristics, memantine possesses wide margin between therapeutic effects and AEs. Similarly, E2730 may be a promising ASM candidate with wide margin between anti‐seizure effects and AEs given that ambient GABA concentration in the brain is relatively low prior to seizure onset then increases during seizures.^20^ E2730, an uncompetitive GAT1 inhibitor, could selectively suppress GAT1 activity under ictal but not physiological conditions in people with epilepsy. In addition, the therapeutic effects of E2730 have been observed in several animal epilepsy models (Table S2). The 6 ‍Hz–44 mA‐stimulated psychomotor seizure mouse model is a well‐known pharmaco‐resistant model for focal epilepsy, for which only a few ASMs show anti‐seizure effects.^21^ In accordance with Barton et al.,^21^ levetiracetam, which was used as a reference ASM, did not show ≥50% efficacy in our experiment. In the pharmaco‐resistant model, E2730 showed a dose‐dependent anti‐seizure effect with an ED_50_ of 17 mg/kg. Amygdala kindling rats are a clinically relevant model of temporal lobe epilepsy.^22^ Levetiracetam was also used as a reference ASM in this model given its previously reported efficacy.^23^ The effective plasma concentration of levetiracetam in people with epilepsy ranged between 10 and 37 μg/mL, which was similar to the range in a rat model of spontaneous recurrent seizures.^24^ Levetiracetam was administered at 25 mg/kg in our study because its plasma concentration was expected to reach the effective range based on pharmacokinetic data of levetiracetam.^25^ E2730 increased the ADT ratio in a dose‐dependent manner in amygdala kindling rats, and the effect size of 10 mg/kg E2730 was comparable to that of 25 mg/kg levetiracetam. The ADT ratios of E2730 10 mg/kg and levetiracetam 25 mg/kg were 129% and 127%, respectively.

The anti‐seizure effect of E2730 for two orphan epilepsy syndromes, FXS and DS, was also evaluated in relevant animal models. In FXS, E/I imbalance caused by functional impairment of inhibitory GABAergic neurons has been reported.^26^, ^27^ Therefore, we hypothesized that E2730 may provide seizure control for FXS by increasing the extracellular GABA concentration. As *Fmr1* KO mice exhibit increased susceptibility to audiogenic seizures,^28^ the effect of E2730 against audiogenic seizures was assessed in audio‐stimulated *Fmr1* KO mice, and our results showed that E2730 suppressed tonic–clonic seizures in this mouse model with an ED_50_ of 17.1 mg/kg. DS is a severe genetic epilepsy syndrome caused by loss‐of‐function mutations in *SCN1A*, a gene encoding the α1 subunit of the Na_v_1.1.^29^ Seizure onset in patients with DS typically begins in infancy, and seizures are often provoked by fever then evolve into status epilepticus.^30^, ^31^ Affected children respond poorly to clinically available ASMs.^32^ The *Scn1a* ^+/−^ mice recapitulate the characteristics of patients with DS.^32^ Previous studies have demonstrated that Na_v_1.1 is predominantly clustered in the axon initial segments of parvalbumin‐positive inhibitory interneurons, and loss of function of Na_v_1.1 in inhibitory interneurons results in E/I imbalance with the reduced inhibitory tone, leading to a greater seizure susceptibility in patients with DS compared with healthy people.^33^, ^34^, ^35^ Therefore, enhancement of GABA signaling may improve seizure control for DS.^32^ Indeed, clonazepam, a positive allosteric modulator of GABA_A_R, is effective in both patients and a mouse model of DS.^30^, ^31^ E2730 significantly increased the seizure threshold for hyperthermia‐induced generalized tonic–clonic seizures in *Scn1a* ^+/−^ mice, supporting our hypothesis that E2730 may be effective against DS by increasing GABAergic inhibitory tone.

There is a technical limitation in the microdialysis assay, which measures extracellular concentrations and cannot distinguish synaptic concentrations from extrasynaptic concentrations. E2730 increases synaptic GABA concentration given that GAT1 is concentrated in presynaptic terminals.^36^ The local elevation of GABA would be diluted before it reached to the microdialysis probe. In contrast, high‐potassium stimulation induces GABA release not only from neurons but also from astrocytes.^37^ Therefore, the GABA increase in the vehicle group (high‐potassium stimulation only) reflects the elevation of both synaptic and extrasynaptic GABA levels. These would be the potential reason why 10 mg/kg of E2730 was not statistically significant in the microdialysis assay, while the 10 mg/kg was effective in some of the seizure models.

In conclusion, E2730 is a novel, selective, uncompetitive GAT1 inhibitor, and may be a potential ASM candidate with a wide margin between anti‐seizure effect and AEs (i.e., motor incoordination) against multiple types of epilepsy, including focal epilepsy, pharmaco‐resistant epilepsy, and orphan epilepsy syndromes. Since the cause of epilepsy is not homogeneous, precision medicine based on causes of each patient is needed. For the precision medicine, a single MOA drug would be an advantage over a drug with multiple MOA because it is easy to customize a combination of drugs each of which has a different MOA related to one of the causes of a patient. Thus, E2730 would potentially provide an additional therapeutic option to treat epilepsy with impaired GABAergic functions.

## CONFLICT OF INTEREST STATEMENT

Kazuyuki Fukushima, Yuji Kazuta, Keisuke Hashimoto, Naoto Watanabe, Yoshiaki Furuya, Ting Wu, Takahisa Hanada, and Katsutoshi Ido are all employees of Eisai Co., Ltd. Hiroyuki Higashiyama, Yoshimasa Ito, and Takashi Kosasa were employees of Eisai Co., Ltd. during these works and now are retired. Delia M. Talos, Yeri Song, Nicholas S. Roberts, and Frances E. Jensen contributed to a part of these studies as sponsored research. We confirm that we have read the Journal's position on issues involved in ethical publication and affirm that this report is consistent with those guidelines.

## Supporting information


Figure S1

Table S1

## Data Availability

The data that support the findings of this study are available from the corresponding author upon reasonable request.
