# Supplementary material for: Discovery of E2730, a novel selective uncompetitive GAT1 inhibitor, as a candidate for anti‐seizure medication
Source: Epilepsia Open. 2023 May 18;8(3):834–45. doi: 10.1002/epi4.12741 (PMC10472371; doi:10.1002/epi4.12741)
Supplement: Supplementary file 1 — Figure S1 [file EPI4-8-834-s001.docx]

# **FIGURE S1. Effects of E2730, tiagabine, and diazepam on GABA-induced current amplitude in hGABA_A_R-expressing cells.**


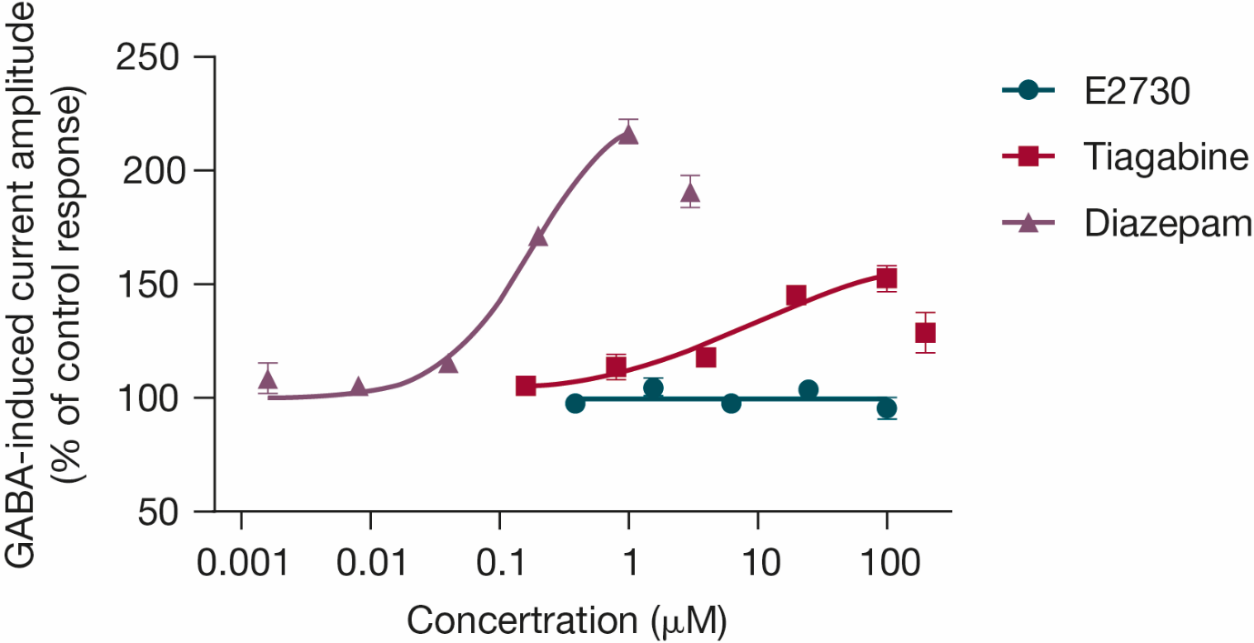


Data represent the mean ± SEM of 3 independent experiments. Control response is GABA (13 μM)-induced current amplitude. Effects of E2730, tiagabine, and diazepam on GABA-induced current amplitude were examined in HEK-293 cells stably expressing hGABA_A_R (containing α1, β3, and γ2 subunits) using whole-cell patch-clamp assay with the automated Qpatch HTX system (Sophion Bioscience, Denmark). Cells in voltage clamp were maintained at -80 mV throughout the experiments following the voltage protocol. Diazepam, a reference ASM of long-acting classical benzodiazepine, potentiated the GABA-induced current amplitude with E_max_ and EC_50_ values of 217.7% (at 1 μM) and 0.15 μM, respectively. Tiagabine also potentiated the GABA-induced current amplitude with E_max_ and EC_50_ values of 153.2% (at 100 μM) and 4.8 μM, respectively. In contrast, E2730 did not affect the GABA-induced current up to 100 μM.

Abbreviations: E_max_, maximum induction effect_;_ EC_50_, concentration achieving 50% of E_max_; GABA, γ-aminobutyric acid; HEK, human embryonic kidney; hGABA_A_R, human GABA_A_ receptor.

# **Figure S2. Effects of E2730 and desipramine on veratridine-induced hNa_v_1.2 activation in hNa_v_1.2-expressing cells.**


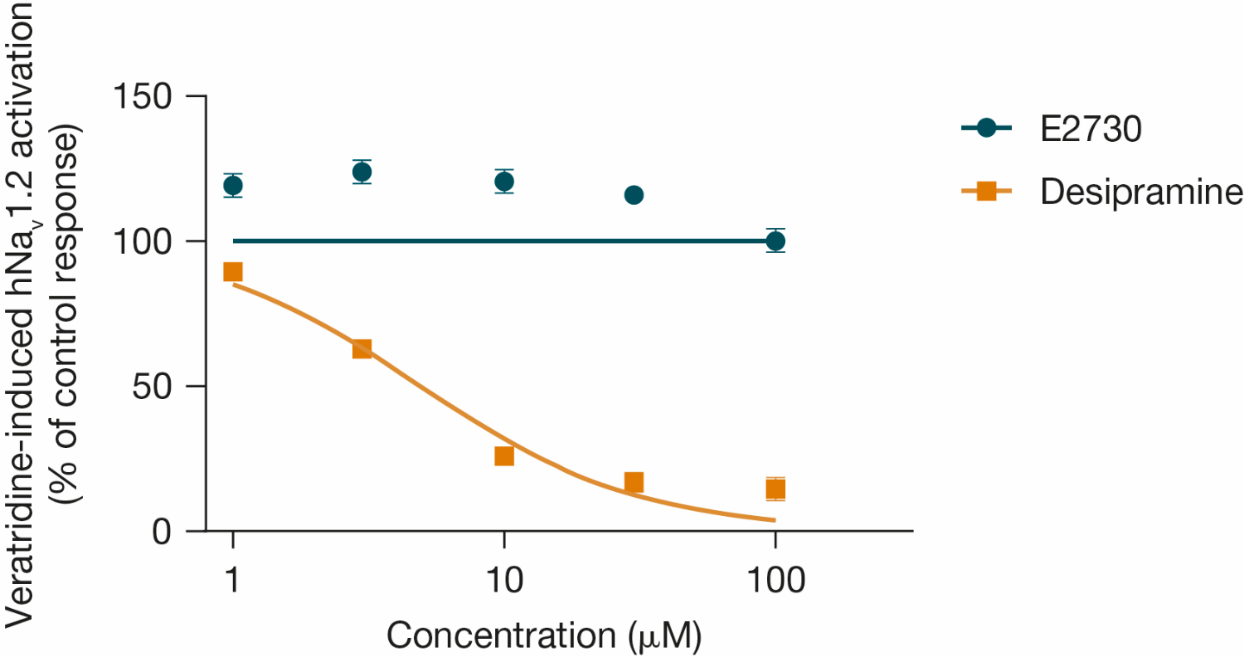


Data represent the mean ± SEM of three independent experiments. Effects of E2730 and desipramine (Sigma-Aldrich, MO) on hNa_v_1.2 activity were evaluated in CHO cells stably expressing hNa_v_1.2 using a fluorescent-based membrane potential assay (Molecular Devices, CA). Activation of hNa_v_1.2 was induced by veratridine (50 μM) and the change of fluorescence (excitation wavelength, 480 nm; emission wavelength, 540 nm) was recorded using Fluorescence Drug Screening System 6000 (Hamamatsu Photonics, Japan). E2730 did not inhibit hNa_v_1.2 activation up to 100 μM. Desipramine, a reference drug of sodium channel inhibitor, inhibited hNa_v_1.2 activation in a concentration-dependent manner with an IC_50_ of 4.9 μM.

Abbreviation: CHO, Chinese hamster ovary; hNa_v_1.2, human Na_v_1.2 voltage-gated sodium channel; IC_50_, 50% inhibitory concentration.
