# Supplementary material for: Discovery of E2730, a novel selective uncompetitive GAT1 inhibitor, as a candidate for anti‐seizure medication
Source: Epilepsia Open. 2023 May 18;8(3):834–45. doi: 10.1002/epi4.12741 (PMC10472371; doi:10.1002/epi4.12741)
Supplement: Supplementary file 2 — Table S1 [file EPI4-8-834-s002.docx]

# **TABLE S1. Panel of E2730 binding assay.**

| **Target** | **Origin** | **Target** | **Origin** | **Target** | **Origin** |
| --- | --- | --- | --- | --- | --- |
| Adenosine, A_1_ | human recombinant | Cholecystokinin CCK_B_ (CCK_2_) | human recombinant | CCR1 | human recombinant |
| Adenosine, A_2A_ | human recombinant | Dopamine, D_1_ | human recombinant | TNF-α | U-937 cells (human) |
| Adenosine, A_3_ | human recombinant | Dopamine, D_2S_ | human recombinant | Histamine, H_1_ | human recombinant |
| α_1_ Adrenergic (non-selective) | rat cerebral cortex | Dopamine, D_3_ | human recombinant | Histamine, H_2_ | human recombinant |
| α_2_ Adrenergic (non-selective) | rat cerebral cortex | Dopamine, D_4.4_ | human recombinant | Melanocortin, MC_4_ | human recombinant |
| β_1_ Adrenergic | human recombinant | Dopamine, D_5_ | human recombinant | Melatonin, MT_1_ (ML_1A_) | human recombinant |
| β_2_ Adrenergic | human recombinant | Endothelin, ET_A_ | human recombinant | Muscarinic, M_1_ | human recombinant |
| Angiotensin, AT_1_ | human recombinant | Endothelin, ET_B_ | human recombinant | Muscarinic, M_2_ | human recombinant |
| Angiotensin, AT_2_ | human recombinant | GABA  (non-selective) | rat cerebral cortex | Muscarinic, M_3_ | human recombinant |
| Benzodiazepine (central) | rat cerebral cortex | Galanin, GAL_1_ | human recombinant | Muscarinic, M_4_ | human recombinant |
| Benzodiazepine (peripheral) | rat heart | Galanin, GAL_2_ | human recombinant | Muscarinic, M_5_ | human recombinant |
| Bombesin, BB (non-selective) | rat cerebral cortex | AMPA | rat cerebral cortex | Neurokinin, NK_1_ | U-373MG cells (human) |
| Bradykinin, B_2_ | human recombinant | Kainate | rat cerebral cortex | Neurokinin, NK_2_ | human recombinant |
| Calcitonin gene related peptide, CGRP | human recombinant | NMDA | rat cerebral cortex | Neurokinin, NK_3_ | human recombinant |
| Cannabinoid, CB_1_ | human recombinant | PDGF | BALB/c 3T3 cells (mouse) | Neuropeptide, Y_1_ | SK-N-MC cells (human) |
| Cholecystokinin CCK_A_ (CCK_1_) | human recombinant | CXCR2 (IL-8B) | human recombinant | Neuropeptide, Y_2_ | KAN-TS cells (human) |

# **TABLE S1. Panel of E2730 binding assay (Continued).**

| **Target** | **Origin** | **Target** | **Origin** | **Target** | **Origin** |
| --- | --- | --- | --- | --- | --- |
| Neurotensin, NT_1_ (NTS_1_) | human recombinant | Purinergic, P2Y | rat cerebral cortex | Vasoactive intestinal peptide, VIP_1_ (VPAC_1_) | human recombinant |
| N neuronal α4β2 | SH-SY5Y cells (human) | Serotonin, 5-HT_1A_ | human recombinant | Vasopressin, V_1a_ | human recombinant |
| δ_2_ Opioid | human recombinant | Serotonin, 5-HT_1B_ | rat cerebral cortex | L-Type Ca^2+^ channel (dihydropyridine) | rat cerebral cortex |
| κ Opioid | rat recombinant | Serotonin, 5-HT_2A_ | human recombinant | L-type Ca^2+^ channel (verapamil) | rat cerebral cortex |
| μ Opioid | human recombinant | Serotonin, 5-HT_2B_ | human recombinant | N-Type Ca^2+^ channel | rat cerebral cortex |
| Nociceptin, NOP (ORL1) | human recombinant | Serotonin, 5-HT_2C_ | human recombinant | K^+^_V_ channel | rat cerebral cortex |
| PAC_1_ (PACAP) | human recombinant | Serotonin, 5-HT_3_ | human recombinant | SK^+^_Ca_ channel | rat cerebral cortex |
| PPARγ | human recombinant | Serotonin, 5-HT_5a_ | human recombinant | Na^+^ channel  (site 2) | rat cerebral cortex |
| Phencyclidine, PCP | rat cerebral cortex | Serotonin, 5-HT_6_ | human recombinant | Cl^-^ channel (GABA-gated) | rat cerebral cortex |
| Prostaglandin E2, EP_2_ | human recombinant | Serotonin, 5-HT_7_ | human recombinant | Norepinephrine Transporter | human recombinant |
| Prostaglandin E2, EP_4_ | human recombinant | Sigma  (non-selective) | cellules Jurkat (human) | Dopamine Transporter | human recombinant |
| Prostacyclin, IP (PGI_2_) | human recombinant | Somatostatin (sst) (non-selective) | AtT-20 cells (mouse) | 5-HT Transporter | human recombinant |
| Purinergic, P2X | rat urinary bladder | Glucocorticoid, GR | IM-9 cells (human) |  |  |

In this binding panel assay, E2730 was tested at 30 and 300 μM. No significant binding was observed at each concentration.

AMPA = α-amino-3-hydroxy-5-methyl-4-isoxazole propionic acid, CCR1 = CC chemokine receptor 1, CXCR2 = CXC chemokine receptor 2, GABA = γ-aminobutyric acid, NMDA = *N*-methyl-d-aspartate, NOP = nociception, ORL1 = nociception receptor, PACAP = pituitary adenylate cyclase activating polypeptide, PDGF = platelet-derived growth factor, PPAR = peroxisome proliferator-activated receptor, TNF = tumor necrosis factor.

# **TABLE S2. Anti-seizure profile of E2730 in animal models.**

| **Model** | **ED_50_ (95% CI), mg/kg** |
| --- | --- |
| Pharmaco-resistant 6 Hz-44 mA psychomotor  seizures (mouse) | E2730: 17 (12, 24)  Levetiracetam: >200 |
| Amygdala kindling (rat) | E2730: 10^a^ (129%^b^)  Levetiracetam: 25^a^ (127%^b^) |
| Audiogenic seizures (*Fmr1* knockout mouse) | E2730: 17.1 (12.2, 21.8) |
| Hyperthermia-induced seizures (*Scn1a*^+/-^ mouse) | E2730: 10^a^ (41.2°C^c^) |

^a^ Minimum effective dose.

^b^ After-discharge threshold ratio at the minimum effective dose.

^c^ Median threshold body temperature for generalized tonic-clonic seizures at the

minimum effective dose.

Abbreviations: CI, confidence interval; ED_50_, 50% effective dose.
